# Urinary Carnosinase-1 Excretion is Associated with Urinary Carnosine Depletion and Risk of Graft Failure in Kidney Transplant Recipients: Results of the TransplantLines Cohort Study

**DOI:** 10.3390/antiox10071102

**Published:** 2021-07-09

**Authors:** Angelica Rodriguez-Niño, Diego O. Pastene, Adrian Post, M. Yusof Said, Antonio W. Gomes-Neto, Lyanne M. Kieneker, M. Rebecca Heiner-Fokkema, Tuba Esatbeyoglu, Gerald Rimbach, Peter Schnuelle, Benito A. Yard, Stephan J. L. Bakker

**Affiliations:** 1 Department of Internal Medicine, University Medical Center Groningen, University of Groningen, 9700 RB Groningen, The Netherlands; a.post01@umcg.nl (A.P.); m.y.said@umcg.nl (M.Y.S.); a.w.gomes.neto@umcg.nl (A.W.G.-N.); l.m.kieneker@umcg.nl (L.M.K.); p.schnuelle@nierenzentrum-weinheim.de (P.S.); s.j.l.bakker@umcg.nl (S.J.L.B.); 2 Department of Nephrology, Endocrinology and Rheumatology (Fifth Department of Medicine), Medical Faculty Mannheim, University of Heidelberg, 68167 Mannheim, Germany; diego.pastene@medma.uni-heidelberg.de (D.O.P.); benito.yard@medma.uni-heidelberg.de (B.A.Y.); 3 Department of Laboratory Medicine, University Medical Center Groningen, University of Groningen, 9713 GZ Groningen, The Netherlands; m.r.heiner@umcg.nl; 4 Institute of Food Science and Human Nutrition, Gottfried Wilhelm Leibniz University Hannover, 30167 Hannover, Germany; esatbeyoglu@lw.uni-hannover.de; 5 Institute of Human Nutrition and Food Science, Christian-Albrechts University of Kiel, 24118 Kiel, Germany; rimbach@foodsci.uni-kiel.de; 6 Center for Renal Diseases, 69469 Weinheim, Germany

**Keywords:** carnosine, carnosinase-1, kidney transplantation, oxidative stress, graft failure

## Abstract

Carnosine affords protection against oxidative and carbonyl stress, yet high concentrations of the carnosinase-1 enzyme may limit this. We recently reported that high urinary carnosinase-1 is associated with kidney function decline and albuminuria in patients with chronic kidney disease. We prospectively investigated whether urinary carnosinase-1 is associated with a high risk for development of late graft failure in kidney transplant recipients (KTRs). Carnosine and carnosinase-1 were measured in 24 h urine in a longitudinal cohort of 703 stable KTRs and 257 healthy controls. Cox regression was used to analyze the prospective data. Urinary carnosine excretions were significantly decreased in KTRs (26.5 [IQR 21.4–33.3] µmol/24 h versus 34.8 [IQR 25.6–46.8] µmol/24 h; *p* < 0.001). In KTRs, high urinary carnosinase-1 concentrations were associated with increased risk of undetectable urinary carnosine (OR 1.24, 95%CI [1.06–1.45]; *p* = 0.007). During median follow-up for 5.3 [4.5–6.0] years, 84 (12%) KTRs developed graft failure. In Cox regression analyses, high urinary carnosinase-1 excretions were associated with increased risk of graft failure (HR 1.73, 95%CI [1.44–2.08]; *p* < 0.001) independent of potential confounders. Since urinary carnosine is depleted and urinary carnosinase-1 imparts a higher risk for graft failure in KTRs, future studies determining the potential of carnosine supplementation in these patients are warranted.

## 1. Introduction

Kidney transplantation is the treatment of choice in patients with end stage renal disease. Compared to treatment by dialysis, it reduces mortality, is cost effective, and increases quality-adjusted life years [1,2,3,4]. However, despite significant progress in immunosuppression and supportive treatment, half of kidney recipients still experience graft failure or die within a decade after transplantation [5]. Thus, identification of patients at risk of early graft failure is decisive in improving graft survival rate. Even though a kidney biopsy is the gold standard for the diagnosis of graft rejection, identification of potentially modifiable risk factors for predicting risk of graft failure might help to improve long-term graft survival.

We and others have previously reported that a polymorphism in the *CNDP1* gene, encoding serum carnosinase-1 (CN1), is associated with lower risk of developing diabetic nephropathy in patients with type 2 diabetes [6,7,8,9,10]. Additionally, in a prospective study of pediatric patients with non-diabetic nephropathies, the *CNDP1* polymorphism was found to correlate with renal survival, particularly in patients with glomerulopathies [11]. Although these studies provide compelling evidence for a protective role of *CNDP1* in renal disease, other studies have failed to replicate these findings [12,13]. Yet, based on the available in vitro and in vivo evidence, the current hypothesis on the role of CN1 in kidney disease puts forward that low serum CN1 concentrations and low enzymatic activity will promote high tissue carnosine concentrations. As carnosine and other histidine-containing dipeptides (HCDs) are endowed with reno-protective properties in the setting of oxidative and glycative stress [6,14], depletion of such HCDs will render the kidney tissue more vulnerable to hyperglycemia or other diseases in which oxidative stress prevails.

The plausibility that CN1 may have a negative effect on the progression of kidney disease is further substantiated by in vivo studies showing that overexpression of CN1 aggravates diabetic nephropathy and results in decreased plasma and kidney carnosine levels [15,16,17]. In contrast, carnosine feeding in diabetic and non-diabetic animal models decreases urinary markers of oxidative and carbonyl stress, reduces proteinuria, and mitigates glomerular damage [15,18,19,20]. Of note, while circulating serum carnosine is difficult to detect as a result of CN1 enzymatic activity, urinary carnosine, which may, to some extent, be expected to reflect serum carnosine, appears to be an easier and reliable estimation [20,21]. Indeed, in humans, urinary carnosine appeared to correlate positively with serum carnosine, and inversely with serum CN1 concentrations [22].

We have recently reported that CN1 is detected in urine of healthy subjects and patients with type 2 diabetes [23]. Our previous studies suggest that high urinary CN1 levels in diabetic and non-diabetic patients with chronic kidney disease (CKD) are associated with renal function decline and the degree of albuminuria [23,24].Whether high urinary CN1 results in low urinary carnosine is currently unknown. If so, it would suggest that urinary CN1 indirectly correlates with the ability of the kidney to protect itself against oxidative and carbonyl stress.

Interstitial fibrosis and tubular atrophy (IFTA) is a chronic, progressive, and irreversible histopathological entity that commonly occurs early after transplantation and is the major cause of late allograft failure [25,26,27]. Oxidative stress is a main contributor to IFTA [28] and is inevitably present in kidney transplant recipients (KTRs) during the early period post-transplantation [29]. In keeping with the reno-protective properties of carnosine and the role of CN1 in the progression of renal disease, the primary aim of this study was to investigate the association of urinary CN1 excretion with graft failure in a large cohort of stable KTRs. Secondly, we assessed whether urinary carnosine excretion differs between KTRs and healthy subjects and, thirdly, whether urinary CN1 is inversely associated with urinary carnosine.

## 2. Materials and Methods

### 2.1. Study Population

In this observational prospective study, adult KTRs (≥18 years old) who visited the outpatient clinic of the University Medical Center Groningen between 2008 and 2011, and with a functional graft of at least 1 year, were invited to participate (TransplantLines Food and Nutrition Biobank and Cohort Study, Clinicaltrials.gov № NCT02811835) [30,31]. Healthy kidney donors who participated in the screening program donation, and whose biomaterials were collected prior to kidney donation, were included as a control group. All participants signed the informed consent. The study protocol was approved by the institutional ethical review board (Medical Ethical Committee 2008/186) adhering to the Declaration of Helsinki. Subjects with missing data on urinary CN1 were excluded, leaving 703 KTRs and 257 healthy subjects eligible for analyses.

### 2.2. Data Collection, Clinical and Laboratory Measurements

Baseline measurements were obtained during a morning visit at the outpatient clinic. Subjects were instructed to collect 24 h urine and to fast overnight prior to the visit. Information on patient’s health status, medication use, and medical history was obtained from medical records. Body composition and hemodynamic parameters were measured according to a strict standardized protocol [31]. Serum parameters including lipid, inflammation, and glucose homeostasis were measured with spectrophotometric-based routine laboratory methods (Roche Diagnostics, Rotkreuz, Switzerland). Urinary malondialdehyde (uMDA) was measured by validated high-performance liquid chromatography on a Jasco system (Jasco GmbH Deutschland, Gross-Umstadt, Germany) after derivatization of urine samples with 2,4-dinitrophenylhydrazine (DNPH) using an ODS2 column (10 cm × 4.6 mm, 3 μm) and a photodiode array detector. The mobile phase consisted of 0.2% acetic acid (v/v) in distilled water and acetonitrile (42:58, v/v). The method was validated in terms of linearity, lower limit of quantification, lower limit of detection, precision, accuracy, recovery, and stability according to the U.S. Food and Drug Administration (FDA) guidelines [32]. Urinary liver-type fatty acid-binding protein (uL-FABP) was measured by ELISA (human uL-FABP assay kit 96 test; CMIC holdings Co, Japan) with a detection limit of 0.036 µg/L, intra-assay variabilities of 3.8% and 2.5%, and inter-assay variabilities of 10.4% and 7.3% based on four replicate measurements of urine samples with uL-FABP concentrations of 2 and 40 µg/L, respectively [33].

Diabetes was diagnosed based on the American Diabetes Association criteria (2017) as having fasting plasma glucose ≥ 7.0 mmol/L, or/and HbA1c ≥ 48 mmol/mol, or the use of antidiabetic medication [34]. Kidney function was assessed by estimation of the glomerular filtration rate (eGFR) based on the Chronic Kidney Disease Epidemiology Collaboration equation with serum creatinine and cystatin C [35]. Proteinuria was diagnosed if total urinary protein excretion was ≥0.5 g/24h. Delayed graft function was defined as oliguria of >7 days, or the need for peritoneal dialysis or hemodialysis. Dietary intake was assessed with a previously validated food frequency questionnaire (FFQ) [36], which inquired about intakes of 177 food items during the past month, considering seasonal variations. Dietary data were converted into daily nutrient intakes using the Dutch Food Composition Table 2006 [37]. Dietary intakes were adjusted for total energy intake (kcal/24 h) according to the residual method [38]. In addition to the FFQ, protein intake was also estimated using the Maroni equation [39]. Smoking behavior and alcohol consumption were assessed with a separate questionnaire [40].

### 2.3. Assessment of Urinary CN1 and Urinary Carnosine

Urinary CN1 concentrations were measured by ELISA as previously described [23,24] and can be found in detail in Appendix A (Appendix A.1, Carnosinase-1 Determination in Urine by ELISA). Measurement of urinary carnosine concentrations was performed as part of amino acid profiling with validated liquid chromatography isotope dilution mass spectrometry and is described in detail in Appendix A (Appendix A.2, Amino Acid Profiling with Liquid Chromatography Isotope Dilution Mass Spectrometry).

### 2.4. Clinical Endpoints

The primary endpoint of this study was death-censored graft failure, which was defined as re-transplantation or return to dialysis. The surveillance system of the outpatient clinic program ensured up-to-date information on KTR status with no loss to follow-up. Endpoints were recorded until September 2015.

### 2.5. Statistical Analyses

Data analyses were performed with SPSS 25.0 software (IBM Corporation) and R software version 3.5.1 (R Foundation for Statistical Computing). Data are presented as mean ± standard deviation (SD) for normally distributed data, median and interquartile range (IQR) for skewed distributions, or absolute number and percentage for nominal data. A two-sided *p* value ˂ 0.05 was considered as statistically significant. Baseline differences between KTRs and healthy kidney donors were assessed with an unpaired *t*-test, Mann–Whitney U test, or chi-square test. A binary logarithmic (log2) transformation was applied to urinary CN1 excretions, urinary CN1 concentrations, and urinary CN1/creatinine ratios and used as such through the entire regression analysis. Logistic regression was performed to assess the association of urinary CN1 concentration with urinary carnosine with subsequent cumulative adjustment for sex, age, and eGFR. The associations of urinary CN1 excretion with baseline characteristics of KTRs were studied by linear regression with subsequent cumulative adjustment for age, sex, and eGFR. Regression coefficients are presented as standardized beta values (St. β) referring to the number of SDs the dependent variable changes per SD increase of the independent variable, allowing comparison of different variables.

#### Prospective Analyses

Cox regression models were employed to investigate the association of urinary CN1 excretions, urinary CN1 concentrations, and urinary CN1/creatinine ratio with risk of graft failure. The Cox regression models were built up in a stepwise fashion to keep the number of predictors in proportion to the number of events. Adjustments were made a priori and for relevant variables identified by linear regression if the *p* value for the association with urinary CN1 excretion was ˂0.05. Crude associations (Model 1) were cumulatively adjusted for basic confounders, i.e., age, sex, body mass index (Model 2), and renal function, i.e., eGFR and proteinuria (Model 3). To prevent overfitting, Model 3 was additionally adjusted for cardiovascular risk factors (Model 4), transplantation-related factors (Model 5), post-transplantation complications (Model 6), and urinary parameters (Model 7). Cardiovascular risk factors were defined as systolic blood pressure, HDL cholesterol, triglycerides, use of antihypertensive treatment, presence of diabetes, and medical history of cardiovascular intervention. Transplantation factors were defined as dialysis vintage, time from transplantation to study baseline, deceased donor, number of transplantations up to baseline, and calcineurin inhibitor use. Post-transplantation complications were defined as delayed graft function, graft rejection, and post-transplant diabetes mellitus. The proportional hazard assumption was checked for urinary CN1 excretions, urinary CN1 concentrations, and urinary CN1/creatinine ratio. Potential interactions of urinary CN1 with age, sex, BMI, eGFR, proteinuria, and urinary urea excretions for the associations with graft failure were checked. To visualize the continuous associations of urinary CN1 with the risk of graft failure, log2-transformed urinary CN1 excretion, urinary CN1 concentration, and urinary CN1/creatinine ratio were individually plotted against the risk of graft failure.

## 3. Results

### 3.1. Urinary Carnosine is Reduced in KTRs Compared to Healthy Donors

We studied 703 stable KTRs (57% male; age 53 ± 13 years old) at a median time of 5.4 [IQR 1.9–12.0] years after transplantation, and 257 healthy kidney donors (44% male; age 54 ± 11 years old). The characteristics of KTRs and healthy donors are depicted in Table 1.

Median urinary CN1 excretions were similar in KTRs and healthy donors (29.4 [17.4–49.2] µg/24 h versus 32.7 [21.6–43.6] µg/24 h; *p* = 0.42). Similarly, there were no differences in median urinary CN1 concentrations and median urinary CN1/creatinine ratios between the studied groups. KTRs with proteinuria had higher urinary CN1 excretions in comparison to KTRs without proteinuria and to healthy subjects (data not shown). Of note, urinary CN1 was below the detection limit in 95 (14%) of KTRs compared to 33 (13%) healthy donors (Table 1).

Data are presented as mean ± SD, percentage, or median [IQR]. *p* value for statistical difference was tested by an independent *t*-test, Mann–Whitney U test, or chi-squared test. * Dietary intakes were adjusted for energy intake according to the residual method. Data were available for 640 KTRs and 173 healthy subjects. Abbreviations: BMI: body mass index, HDL: high-density lipoprotein, LDL: low-density lipoprotein, eGFR: estimated glomerular filtration rate, NT-proBNP: N-terminal pro-brain natriuretic peptide, hsCRP: high-sensitivity C reactive protein, h: hour.

Interestingly, urinary carnosine excretion was significantly lower in KTRs (26.5 [21.4–33.3] µmol/24 h versus 34.8 [25.6–46.8] µmol/24 h; *p* < 0.001) compared to the healthy group. Likewise, urinary carnosine concentration and urinary carnosine/creatinine ratios were lower in KTRs (all *p* < 0.001). In line with this, the proportion of subjects with urinary carnosine below the detection limit was higher in the KTRs compared to healthy controls (66% versus 37%; *p* < 0.001).

KTRs had a higher BMI in comparison to the healthy group. As anticipated, eGFR was significantly lower in KTRs (45 ± 18 mL/min/1.73 m^2^ versus 92 ± 16 mL/min/1.73 m^2^; *p* < 0.001). Accordingly, serum creatinine levels (125 [99–160] µmol/L versus 72 [64–81] µmol/L; *p* < 0.001) and the proportion of subjects with proteinuria (22% versus 0.5%; *p* < 0.001) were also significantly higher in KTRs. Additionally, systolic and diastolic blood pressures were higher in KTRs. Furthermore, KTRs had higher levels of triglycerides, N-terminal pro-brain natriuretic peptide (NT-proBNP), and high-sensitivity C-reactive protein (hs-CRP) (all *p* < 0.001). In the KTR group, there were more subjects with diabetes and HbA1c levels were also higher (*p* < 0.001). The diet of the KTRs was characterized by lower energy intake and lower FFQ-derived protein intake compared to healthy controls (*p* < 0.001). KTRs had also lower creatinine excretion per 24 h (*p* < 0.001) (Table 1).

### 3.2. Urinary CN1 is Inversely Associated with Urinary Carnosine in KTRs

The low excretions per 24h and per mmol creatinine and low concentrations of urinary carnosine in KTRs prompted us to explore the association between urinary CN1 and urinary carnosine (Table 2). In KTRs, unadjusted logistic regression analyses revealed that urinary CN1 concentrations were associated with increased risk for undetectable carnosine in urine (OR 1.24, 95%CI [1.06–1.45]; *p* = 0.007). This inverse association was independent of age and sex, but lost significance after further adjustment for eGFR (OR 0.96, 95%CI [0.80–1.14]; *p* = 0.60). No significant associations were found between urinary carnosine and urinary CN1 in the healthy group (all *p* > 0.05). Of note, in KTRs, urinary carnosine excretions were associated with meat intake (St. β = 0.12, *p* = 0.001).

Unadjusted and multivariable-adjusted logistic regression analyses were performed to investigate the association of urinary carnosine with urinary CN1 concentrations in KTRs with adjustment for potential confounders. Urinary CN1 concentrations were log2-transformed for analyses. Model 1: crude association, Model 2: adjusted for sex and age, Model 3: adjusted as for Model 2 and for eGFR. Odds ratios and 95% confidence intervals were calculated with urinary carnosine as a dichotomous variable according to its detection cut-off in urine. Abbreviations: OR: odds ratio, CI: confidence interval, CN1: carnosinase-1, eGFR: estimated glomerular filtration rate, KTRs: kidney transplant recipients.

### 3.3. Urinary CN1 Excretion Is Associated with Urinary Oxidative Stress Markers

The regression coefficients for the association of baseline characteristics and urinary CN1 excretions in KTRs are shown in Table 3. In univariable analyses, urinary CN1 excretion was positively associated with urinary CN1 concentration, urinary CN1/creatinine ratio, systolic blood pressure, NT-proBNP, triglycerides, serum creatinine, proteinuria, graft rejection, FFQ-derived total protein intake, animal protein intake, lactate dehydrogenase (LDH), urinary sodium excretion, urinary potassium excretion, urinary urea excretion, Maroni formula-derived protein intake, use of antihypertensive medication, use of diuretics, uMDA excretion, and uL-FABP excretion. Moreover, urinary CN1 excretion was inversely associated with eGFR, creatinine clearance, and sodium (all *p* < 0.05) (Table 3, Model 1).

The adjustment for age, sex, and eGFR revealed an association of urinary CN1 excretion with HDL cholesterol and urinary creatinine excretion. In contrast, the association of urinary CN1 excretion with triglycerides, serum creatinine, graft rejection, animal protein intake, LDH, use of diuretics, and antihypertensive medication was no longer significant after adjustments (all *p* > 0.05).

Furthermore, adjustment for age, sex, and eGFR strengthened the association of urinary CN1 excretion with creatinine clearance, FFQ-derived protein intake, urinary sodium excretion, urinary potassium excretion, urinary urea excretion, and Maroni formula derived protein intake (all *p* < 0.05). The associations of urinary CN1 excretion with NT-proBNP, proteinuria, sodium, and uL-FABP excretion weakened, yet remained significant after adjustment for age, sex, and eGFR (Table 3, Model 2).

### 3.4. Urinary CN1 Is Associated with High Risk of Graft Failure in KTRs

During median follow-up of 5.3 [4.5–6.0] years, 84 (12%) KTRs developed graft failure. Of these, 62 (74%) developed chronic rejection, and eight (10%) had recurrence of the primary disease. KTRs who experienced graft failure had higher median urinary CN1 excretions (42.2 [27.9–92.6] µg/24 h versus 27.9 [16.0–45.2] µg/24 h; *p* < 0.001) compared to KTRs with preserved renal grafts. This was also the case for urinary CN1 concentrations (21.7 [13.8–36.5] µg/L versus 11.7 [7.8–19.1] µg/L; *p* < 0.001) and urinary CN1/creatinine ratios (4.5 [2.4–8.7] µg/mmol versus 2.5 [1.4–4.2] µg/mmol; *p* < 0.001). In line with this, lower urinary carnosine excretions were found in KTRs experiencing graft failure (23.5 [17.8–27.1] µmol/24 h versus 27.1 [22.1–34.4] µmol/24h; *p* < 0.001) in comparison to KTRs with preserved renal grafts. Similar differences were found for urinary carnosine concentrations and urinary carnosine/creatinine ratios (all *p* < 0.001).

Model 1: crude association, Model 2: crude with adjustment for age, sex, and eGFR. Urinary CN1 was log2-transformed for analysis. Regression coefficients are given as standardized beta values (St. β). Dietary intake was adjusted for energy intake through the residual method. Abbreviations: BMI: body mass index, NT-proBNP: N-terminal pro-brain natriuretic peptide, CVA: cerebrovascular accident, TIA: transient ischemic attack, HDL: high-density lipoprotein, LDL: low-density lipoprotein, HLA: human leukocyte antigen, eGFR: estimated glomerular filtration rate, LDH: lactate dehydrogenase, RAAS: renin–angiotensin–aldosterone system, hsCRP: high-sensitivity C-reactive protein, uMDA: urinary malondialdehyde, uL-FABP: urinary liver-type fatty acid-binding protein.

Prospective analyses of the association between urinary CN1 excretions, CN1 concentrations, and CN1/creatinine ratios with death-censored graft failure are shown in Table 4. The proportional hazard assumption was checked for urinary CN1 excretions, concentrations, and creatinine ratios (all *p* > 0.05). Cox regression analyses revealed an association of log_2_ urinary CN1 excretions (HR 1.73, 95%CI [1.44–2.08]; *p* < 0.001), log_2_ urinary CN1 concentrations (HR 2.05, 95%CI [1.69–2.49]; *p* < 0.001), and log_2_ urinary CN1/creatinine ratio (HR 1.76, 95%CI [1.48–2.09]; *p* < 0.001) with graft failure (Model 1). These associations remained materially unchanged after adjustment for age, sex, and BMI (Model 2). After cumulative adjustment for eGFR and proteinuria, the associations of urinary CN1 excretion (HR 1.24, 95%CI [1.03–1.51]; *p* = 0.026), urinary CN1 concentration (HR 1.36, 95%CI [1.11–1.67]; *p* = 0.003), and urinary CN1/creatinine ratio (HR 1.32, 95% CI [1.09–1.61]; *p* = 0.005) with graft failure somewhat weakened, though they remained significant (Model 3). Moreover, these associations remained independent of additional adjustment for cardiovascular risk factors (Model 4), for transplantation-related parameters (Model 5), for transplantation complications (Model 6), and for urinary parameters (Model 7) (Table 4). No significant interactions with age, sex, BMI, eGFR, proteinuria, and urinary urea excretions were found for the associations of urinary CN1 with graft failure (*p* > 0.05).

Cox regression analyses were performed to assess the associations of log2-transformed urinary CN1 excretion, urinary CN1 concentration, and urinary CN1 creatinine ratio with death-censored graft failure (number of events = 84) in KTRs (n = 703). Model 1: crude associations. Multivariable Model 2: adjusted for basic confounders (age, sex, and BMI). Multivariable Model 3 as for Model 2 with additional adjustment for eGFR and proteinuria. Subsequently, additive adjustments were performed based on variables already adjusted for in Model 3. Model 4: further adjustment for cardiovascular risk factors (systolic blood pressure, HDL cholesterol, triglycerides, antihypertensive medication usage, diabetes mellitus, and medical history of cardiovascular intervention). Model 5 involved further adjustment for transplantation-related factors (dialysis vintage, time from transplantation to baseline, donor type, need for re-transplantation up to baseline, and use of calcineurin inhibitors). Model 6: further adjusted for post-transplantation complications (delayed graft function, graft rejection, and post-transplant diabetes mellitus) and Model 7 involved further adjustment for urinary parameters (urinary sodium, urinary potassium, and urinary urea). Abbreviations: CN1: carnosinase-1, HR: hazard ratio, CI: confidence interval, eGFR: estimated glomerular filtration rate, HDL: high-density lipoprotein.

**Figure 1:**
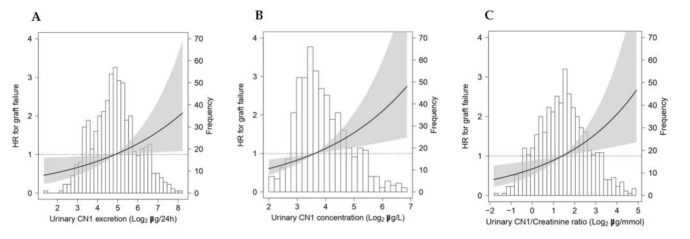
Urinary CN1 and Risk of Graft Failure in KTR. Association of urinary CN1 with death-censored graft failure in KTRs. Continuous association of urinary CN1 excretion A, urinary CN1 concentration B, and urinary CN1/creatinine ratio C with death-censored graft failure in KTRs. Urinary CN1 excretion, urinary CN1 concentrations, and urinary CN1/creatinine ratios were log_2_ -transformed for prospective analyses. The histograms depict the distribution. The black line shows the adjusted hazard ratio (HR), and the gray areas correspond to the 95% confidence interval (CI). X-axis represents urinary CN1 excretion and concentration or creatinine ratios, and the y-axis represents the estimated hazard ratio relative to the mean urinary CN1 excretion, concentration, or creatinine ratio as reference values. The adjusted association was adjusted for age, sex, BMI, eGFR, and proteinuria (Model 3). *p* values were 0.02, 0.003, and 0.005, respectively. Urinary CN1 concentration values below detection level are not shown. Abbreviations: HR: hazard ratio, CN1: carnosinase-1.

The associations of log_2_-transformed urinary CN1 excretion, urinary CN1 concentration, and urinary CN1/creatinine ratio as continuous variables with graft failure are shown in Figure 1.

## 4. Discussion

The key findings of the present study are as follows: urinary carnosine levels are depleted in KTRs in comparison to healthy controls. Secondly, urinary CN1 concentrations are inversely associated with urinary carnosine in the KTR population, although this inverse association appears to be mediated by renal function. Thirdly, high urinary CN1 is independently and positively associated with risk of late graft failure in KTRs. These observations hold true for the analyses of urinary excretions, concentrations, and creatinine/ratios.

Although primarily synthesized in the liver, CN1 is also expressed in the kidney [6,8,41]. Like other secreted proteins, CN1 contains a signal peptide which directs the protein to the secretory pathway [42]. While serum CN1 concentrations depend mostly on the amount of CN1 synthesized by the liver, urinary CN1 concentrations in healthy individuals may reflect local renal CN1 synthesis as the dimeric CN1 protein is too large (130 kD) to cross the glomerular filtration barrier (cut-off ~50 kD) [43]. Under circumstances of an impaired glomerular filtration barrier, CN1 might be filtered, thereby reaching the urinary compartment [23,24]. Of note, in our previous studies and in the current one, we did not detect any CN1 enzymatic activity in urine [23,24]. Owing to the urine-concentrating capacity of the kidney, which is based on the maintenance of a very high urea concentration gradient, interstitial urea concentrations in mammalian renal cells at some segments of the nephron (inner medulla) can reach very high levels (>1.0 M) [44,45]. In addition, urea concentrations are often very high in urine (~0.28 M) [46]. These high intracellular and urine urea concentrations may denature proteins by affecting the secondary and tertiary structure of proteins, thereby altering their enzymatic function. This might explain the absence of CN1 enzymatic activity in the urine. Yet, formal proof of this phenomenon is currently lacking and could be assessed in future studies.

Although urinary CN1 levels did not differ between the healthy group and the KTRs, an inverse association between urinary CN1 concentrations and urinary carnosine was found in the latter group. This finding might indirectly raise the question whether CN1 activity could be enhanced in KTRs. This speculation is further supported by the finding that the relative proportion of urinary carnosine below the detection limit was higher in KTRs (66% versus 37%; *p* < 0.001) (Table 1). Moreover, as under conditions of high oxidative stress, e.g., diabetes and aging, the efficiency of CN1 in cleaving carnosine is increased [47,48,49], this might also be the case for KTRs, who have been shown to inevitably experience increased oxidative stress [50]. In line with this, our study also disclosed a positive and strong association between the oxidative stress markers, i.e., uMDA and L-FABP, with urinary CN1 excretion, making it likely that overall CN1 activity is higher in KTRs. In the study of Zhou et al., an association between low serum carnosine and high serum CN1 concentrations and enzymatic activity was found exclusively in patients with diabetes and severe renal damage. This was accompanied by a positive association between serum CN1 concentrations and the renal expression of oxidative stress markers, i.e., 4-HNE (4-hydroxynonenal) and 8-OHdG (8-hydroxynonenal) [51]. The findings from this study support the hypothesis that carnosine depletion resulting from high CN1 expression might be associated with increased renal damage.

Carnosine is predominantly present in skeletal muscle. As a dietary source, it is abundantly present in meat, with the highest concentrations being found in beef, mutton, pork, and to a lesser extent in poultry [14,52]. It is unlikely that the difference in urinary carnosine levels was due to differences in total meat or red meat intake, as these were similar among KTRs and the healthy group. Since “The Dutch Food Composition Table” does not include information on carnosine content, the exact contribution of the diet to urinary carnosine could not be addressed herein. Furthermore, it might also be the case that the relative contribution of meat intake to urinary carnosine levels varies under healthy and kidney disease conditions. Being freely filtered at the glomerulus, carnosine is partially reabsorbed by high-affinity oligopeptide transporters PEPT1 and PEPT2 in the proximal tubules [53] where it might exert beneficial reno-protective effects [54]. Although the relation between carnosine and kidney function is not well characterized, it is likely that renal clearance of carnosine might be affected by kidney function. Consequently, circulating carnosine in patients with impaired kidney function might be hydrolyzed to a large extent by serum CN1 before reaching the kidney. This assumption is supported by the finding that the inverse association of CN1 and carnosine disappeared after adjustment for eGFR. Future in vitro and in vivo studies manipulating carnosine and CN1 levels are warranted to further establish a potential link between high CN1 activity, low renal carnosine concentrations, oxidative stress, and renal damage.

We have previously shown in cross-sectional studies that urinary CN1 levels are positively associated with albuminuria and inversely with kidney function [23,24]. In agreement with this, the current study also shows that urinary CN1 excretion is likely affected by renal function, reflected by the strong association of urinary CN1 with eGFR and proteinuria. Furthermore, our findings demonstrate that urinary CN1 is associated with risk of graft failure in KTRs. This association was found to be independent of proteinuria and renal function. It is noteworthy that adjustments for previously described determinants of graft loss [55] caused a slight weakening, leaving a significant association of urinary CN1 with graft failure. Along these lines, previous studies have suggested that high CN1 levels are a risk factor for the development of nephropathy in patients with diabetes [6].

Despite the high impact of modern immunosuppressive and overall supportive therapy, patients undergoing kidney transplantation have limited long-term graft survival [56]. One of the contributing mechanisms to renal dysfunction is oxidative stress, reflected by high levels of reactive oxygen species (ROS) and inflammatory markers [57,58]. The assumption that CN1 enzymatic activity might be higher in KTRs as compared to healthy controls is supported by previous reports suggesting that oxidative stress enhances CN1 activity [47,48]. Moreover, urinary CN1 excretions are higher in KTRs who experienced graft failure, which together with a presumably increased CN1 activity in renal tissue may have a large impact on urinary carnosine levels. Hence, high urinary CN1 might render the kidney vulnerable to oxidative and carbonyl stress. The significance of our study lies in the fact that urinary CN1 could potentially be a biomarker of the overall inflammatory state of the kidney through carnosine availability. Future studies in other populations at risk of an inflammatory milieu are warranted.

Carnosine is generally considered as a bioactive food component with potential health benefits. Several physiological roles have been ascribed to carnosine, including high intracellular pH buffer capacity and protection against oxidative and carbonyl stress [59,60,61]. Additional potential mechanisms of the action of carnosine include an overall lowering of chronic low-grade inflammation and acceleration of wound healing by promoting the expression of growth factors and cytokines involved in reparative processes [62]. In animal models of renal ischemia reperfusion, carnosine treatment was associated with attenuation of ischemia reperfusion-induced renal dysfunction [63,64]. Furthermore, in a recent randomized control trial, oral carnosine supplementation resulted in a significant improvement of oxidative stress, glycemic control, and renal function in pediatric patients with diabetic nephropathy [65]. Whether carnosine supplementation in KTRs might have beneficial effects remains to be addressed in future interventional studies. Alternatively, the use of selective CN1 inhibitors [66] or carnosine analogs resistant to CN1 activity [67] might be considered as these compounds have shown promising results in preclinical studies in diabetic models.

Although our study has several key strengths, e.g., large sample size and a comprehensive collection of parameters that enables adjustment for multiple potential confounders, this study suffers from the usual inherent limitations of observational studies, which do not allow for establishing causative links or underlying mechanisms. Furthermore, we did not investigate the association of urinary carnosine with key inflammatory markers such as IL-6 and TNF-α and this could be assessed in future studies. In addition, the population of this study consisted of Caucasian subjects, which calls for caution in case of extrapolation to other ethnicities.

## 5. Conclusions

In conclusion, high urinary CN1 levels are associated with low graft survival in KTRs, who in turn have low urinary carnosine levels. An adverse impact of high CN1 activity seems very likely, since CN1 regulates carnosine and other histidine-containing dipeptides directly involved in tissue protection. As a result, low carnosine might render the kidney vulnerable to oxidative and carbonyl stress. Urinary CN1 could potentially be a marker of the overall inflammatory state of the kidney.

## Figures and Tables

**Table 1:** Baseline characteristics of KTRs compared to healthy subjects.

Variable	KTRs	Healthy Subjects	
	*n* = 703	*n* = 257	*p*-Value
**Urinary CN1**			
CN1 excretion, µg/24 h	29.4 [17.4–49.2]	32.7 [21.6–43.6]	0.42
CN1 concentration, µg/L	12.3 [8.1–20.9]	13 [10.5–16.5]	0.72
CN1/creatinine ratio, µg/mmol	2.7 [1.5–4.6]	2.6 [1.6–3.7]	0.09
Below detection limit (<4 µg/L), n (%)	95 (14)	33 (13)	0.76
**Urinary carnosine**			
Carnosine excretion, µmol/24 h	26.5 [21.4–33.3]	34.8 [25.6–46.8]	<0.001
Carnosine concentration, µmol/L	9.9 [9.9–11.5]	11.4 [9.9–19.5]	<0.001
Carnosine/creatinine ratio, µmol/mmol	2.3 [1.8–3.2]	2.8 [2.1–3.8]	<0.001
Below detection limit (<10 µmol/L), n (%)	465 (66)	96 (37)	<0.001
**Demographics**			
Male n, (%)	399 (57)	114 (44)	0.001
Age, years	53.0 ± 13	53.8 ± 11	0.34
**Body composition**			
BMI, kg/m²	26.7 ± 4.8	26.0 ± 3.5	0.02
Waist circumference, cm	98 ± 15	91.3 ± 10.4	<0.001
**Cardiovascular**			
Systolic blood pressure, mmHg	136 ± 17.5	127 ± 13.1	<0.001
Diastolic blood pressure, mmHg	83 ± 11	77 ± 9	<0.001
Mean arterial pressure, mmHg	107 ± 15	96 ± 16.1	<0.001
Heart rate, bpm	69 ± 12	67 ± 10	0.02
**Lipids**			
Total cholesterol, mmol/L	5.1 ± 1.1	5.4 ± 1.0	0.002
HDL cholesterol, mmol/L	1.4 ± 0.5	1.6 ± 0.5	0.02
LDL cholesterol, mmol/L	3.0 ± 0.9	3.8 ± 1.0	<0.001
Triglycerides, mmol/L	1.7 [1.3–2.3]	1.2 [0.9–1.6]	<0.001
**Glucose homeostasis**			
Diabetes mellitus, n (%)	168 (24)	13 (5)	<0.001
Glucose, mmol/L	5.3 [4.8–6.0]	5.3 [5.0–5.7]	0.22
HbA1c, mmol/mol	41.9 ± 8.9	37.6 ± 3.9	<0.001
**Renal function**			
Serum creatinine, µmol/L	125 [99–160]	72 [64–81]	<0.001
eGFR, ml/min/1.73 m²	45 ± 18	92 ± 16	<0.001
Creatinine clearance, ml/min	66 ± 27	127 ± 40	<0.001
Proteinuria, n (%)	157 (22)	1 (0.5)	<0.001
Dietary intakes *			
Energy intake, kcal/24 h	2170 ± 639	2295 ± 738	0.03
Total protein intake g/24 h	82 ± 12	85±13	0.01
Animal protein intake g/24 h	51 ± 13	52 ± 13	0.17
Total meat intake g/24 h	96 [74–116]	94 [72–115]	0.71
Red meat intake g/24 h	82 [59–103]	80 [60–106]	0.76
**Serum parameters**			
NT-proBNP, ng/L	255 [108–625]	40 [23–68]	<0.001
Sodium, mmol/L	141 ± 2.8	142 ± 2.0	<0.001
Potassium mmol/L	4.0 ± 0.5	3.9 ± 0.3	<0.001
hsCRP, mg/L	1.6 [0.7–4.6]	1.1 [0.6–2.3]	<0.001
**Urinary parameters**			
Urea excretion, mmol/24 h	384 [308–456]	392 [336–463]	0.09
Creatinine excretion, mmol/24 h	11.6 ± 3.5	13.3 ± 4.4	<0.001
**Medication use**			
Antihypertensives, n (%)	620 (88)	29 (11)	<0.001

Data are presented as mean ± SD, percentage, or median [IQR]. *p* value for statistical difference was tested by an independent *t*-test, Mann–Whitney U test, or chi-squared test. * Dietary intakes were adjusted for energy intake according to the residual method. Data were available for 640 KTRs and 173 healthy subjects. Abbreviations: BMI: body mass index, HDL: high-density lipoprotein, LDL: low-density lipoprotein, eGFR: estimated glomerular filtration rate, NT-proBNP: N-terminal pro-brain natriuretic peptide, hsCRP: high-sensitivity C reactive protein, h: hour.

**Table 2:** Association of urinary carnosine with urinary CN1 concentrations in KTRs (*n* = 703).

Variable	Model 1		Model 2		Model 3	
	OR [95%CI]	*p*-Value	OR [95%CI]	*p*-Value	OR [95%CI]	*p*-Value
Urinary CN1 concentrations, µg/L	1.24 [1.06–1.45]	0.007	1.26 [1.07–1.48]	0.006	1.05 [0.88–1.25]	0.60
Sex, male			1.60 [1.12–2.27]	0.009	1.65 [1.14–2.40]	0.008
Age, years			1.04 [1.02–1.05]	˂0.001	1.03 [1.02–1.05]	˂0.001
eGFR, ml/min/1.73²					0.96 [0.95–0.97]	˂0.001

Unadjusted and multivariable-adjusted logistic regression analyses were performed to investigate the association of urinary carnosine with urinary CN1 concentrations in KTRs with adjustment for potential confounders. Urinary CN1 concentrations were log2-transformed for analyses. Model 1: crude association, Model 2: adjusted for sex and age, Model 3: adjusted as for Model 2 and for eGFR. Odds ratios and 95% confidence intervals were calculated with urinary carnosine as a dichotomous variable according to its detection cut-off in urine. Abbreviations: OR: odds ratio, CI: confidence interval, CN1: carnosinase-1, eGFR: estimated glomerular filtration rate, KTRs: kidney transplant recipients.

**Table 3:** Baseline characteristics of KTRs and regression coefficients of the association with urinary CN1 excretions.

Variable	KTR	Model 1		Model 2	
	*n* = 703	St. β	*p*-Value	St. β	*p*-Value
**Urinary CN1**					
CN1 excretion, µg/24h	29.4 [17.4–49.2]	-	-	-	-
CN1 concentration, µg/L	12.3 [8.1–20.9]	0.91	<0.001	0.91	<0.001
CN1/creatinine ratio, µg/mmol	2.7 [1.5–4.6]	0.92	<0.001	0.98	<0.001
**Demographics**					
Male, n (%)	399 (57)	0.02	0.56	0.01	0.72
Age, years	53.0 ± 13	0.03	0.47	0.003	0.94
**Body composition**					
Body surface area, m²	1.94 ± 0.22	−0.01	0.71	−0.03	0.46
BMI, kg/m²	26.7 ± 4.8	0.01	0.89	−0.02	0.65
Waist circumference, cm	98 ± 15	0.003	0.94	−0.05	0.17
**Lifestyle**					
Current smoker, n (%)	83 (12)	0.016	0.89	−0.001	0.98
Alcohol intake, g/24h	2.6 [0.03–11.1]	−0.02	0.56	−0.01	0.78
**Cardiovascular**					
Systolic blood pressure, mmHg	136 ± 17.5	0.09	0.02	0.07	0.09
Diastolic blood pressure, mmHg	83 ± 11	0.002	0.96	−0.01	0.91
Heart rate, bpm	69 ± 12	0.03	0.49	0.03	0.47
NT-proBNP, ng/L	255 [108–625]	0.21	<0.001	0.13	0.01
Hypertension, n (%)	620 (88)	0.06	0.13	0.04	0.22
**Cardiovascular history**					
CVA and/or TIA, n (%)	41 (6)	−0.01	0.77	−0.02	0.60
Myocardial infarction, n (%)	35 (5)	−0.004	0.91	−0.01	0.66
Cardiovascular intervention, n (%)	68 (10)	0.07	0.05	0.05	0.16
**Lipids**					
Total cholesterol, mmol/L	5.1 ± 1.1	0.07	0.06	0.05	0.18
HDL cholesterol, mmol/L	1.4 ± 0.5	0.06	0.13	0.11	0.006
LDL cholesterol, mmol/L	3.0 ± 0.9	0.05	0.16	0.04	0.33
Triglycerides, mmol/L	1.7 [1.3–2.3]	0.08	0.04	0.02	0.63
**Glucose homeostasis**					
Diabetes mellitus, n (%)	168 (24)	0.06	0.11	0.05	0.18
Glucose mmol/L	5.3 [4.8–6.0]	0.0001	0.99	0.01	0.84
HbA1c, mmol/mol	41.9 ± 8.9	0.03	0.48	0.03	0.42
**Renal function**					
Serum creatinine, µmol/L	125 [99–160]	0.20	<0.001	−0.02	0.83
eGFR, ml/min/1.73 m²	45.1 ± 18.7	−0.24	<0.001	−0.24	<0.001
Creatinine clearance, mL/min	66 ± 27	−0.12	0.002	−0.19	0.002
Proteinuria, n (%)	157 (22)	0.43	<0.001	0.40	<0.001
**Transplantation related**					
Dialysis vintage, months	27 [10–51]	0.02	0.54	0.01	0.80
Time since transplantation, years	5.4 [1.9–12.0]	0.03	0.44	0.05	0.23
Transplantation up to baseline, n (%)		0.05	0.16	0.04	0.24
First transplantation	633 (90)				
Re-transplantation	69 (10)				
Age donor, years	43 ± 15.4	0.004	0.92	−0.07	0.07
Sex donor, n (% male)	356 (51)	−0.04	0.30	−0.07	0.06
Donor type, living, n (%)	239 (34)	−0.04	0.30	−0.015	0.71
Cold ischemia time, hours	15 [3–21]	0.03	0.41	0.010	0.79
Warm ischemia, minutes	40 [33–50]	−0.02	0.63	−0.03	0.44
Calcineurin inhibitors, n (%)	381 (56)	0.03	0.52	−0.04	0.29
Proliferator inhibitor, n (%)	567 (84)	−0.07	0.09	−0.03	0.40
Prednisolone dosage, n (%)		0.05	0.21	0.05	0.18
≤7.5 mg/24 h	284 (40)				
˂7.5 mg/24 h	419 (60)				
HLA-I n (%)	106 (15)	0.03	0.44	0.004	0.92
HLA-II n (%)	121 (17)	0.04	0.28	0.01	0.76
Graft rejection up to baseline, n (%)	188 (27)	0.08	0.04	0.05	0.20
Post-transplant diabetes mellitus	131 (19)	0.04	0.34	0.03	0.44
Delayed graft function	52 (7)	0.0001	0.99	−0.03	0.44
**Dietary intake patterns**					
Energy intake, kcal/24 h	2170 ± 639	−0.05	0.26	−0.02	0.62
Total protein intake, g/24 h	82 ± 12	0.10	0.01	0.12	0.004
Plant protein intake, g/24 h	31 ± 6	0.04	0.37	0.07	0.12
Animal protein intake, g/24 h	51 ± 13	0.08	0.04	0.08	0.05
Meat intake, g/24 h	95 [74–116]	0.01	0.28	0.01	0.26
**Serum parameters**					
LDH, U/L	198 [170–232]	0.11	0.005	0.06	0.15
Sodium, mmol/L	141 ± 2.8	−0.14	<0.001	−0.12	0.001
Potassium, mmol/L	4.0 ± 0.5	0.037	0.335	−0.040	0.314
**Urinary parameters**					
Sodium excretion, mmol/24 h	146 [114–190]	0.10	0.01	0.14	<0.001
Potassium excretion, mmol/24 h	70.1 [ 55.4–87.2]	0.13	< 0.001	0.19	<0.001
Urea excretion, mmol/24 h	384 [308–456]	0.13	0.001	0.19	<0.001
Maroni protein intake, g/kg bw/24 h	85.3 ± 20.6	0.12	0.003	0.18	<0.001
Creatinine excretion, µmol/24 h	11.6 ± 3.4	0.04	0.30	0.12	0.01
**Medication use, n (%)**					
RAAS blockage	341 (49)	0.03	0.45	0.01	0.90
Antihypertensives	620 (88)	0.11	0.003	0.07	0.08
Diuretic	285 (41)	0.10	0.008	0.03	0.39
**Oxidative stress and inflammation**					
hsCRP, mg/L	1.6 [0.7–4.6]	0.04	0.27	0.02	0.68
uMDA excretion, µmol/24 h	9.7 [5.8–15.7]	0.11	0.003	0.11	0.003
Plasma MDA, µmol/L	2.5 [1.9–3.7]	−0.04	0.27	−0.02	0.52
uL-FABP excretion, µg/24 h	2.1 [0.9–7.4]	0.29	<0.001	0.24	<0.001
**Primary kidney disease**					
Primary glomerular disease	198 (28)	0.05	0.20	0.05	0.18
Glomerulonephritis	54 (8)	−0.03	0.51	−0.02	0.58
Tubulointerstitial disease	83 (12)	0.002	0.97	0.03	0.43
Polycystic renal disease	146 (21)	−0.04	0.30	−0.07	0.05
Dysplasia and hypoplasia	28 (4)	0.04	0.36	0.05	0.21
Renovascular disease	40 (6)	−0.02	0.63	−0.02	0.54
Diabetic nephropathy	36 (5)	0.04	0.28	0.02	0.57

Model 1: crude association, Model 2: crude with adjustment for age, sex, and eGFR. Urinary CN1 was log2-transformed for analysis. Regression coefficients are given as standardized beta values (St. β). Dietary intake was adjusted for energy intake through the residual method. Abbreviations: BMI: body mass index, NT-proBNP: N-terminal pro-brain natriuretic peptide, CVA: cerebrovascular accident, TIA: transient ischemic attack, HDL: high-density lipoprotein, LDL: low-density lipoprotein, HLA: human leukocyte antigen, eGFR: estimated glomerular filtration rate, LDH: lactate dehydrogenase, RAAS: renin–angiotensin–aldosterone system, hsCRP: high-sensitivity C-reactive protein, uMDA: urinary malondialdehyde, uL-FABP: urinary liver-type fatty acid-binding protein.

**Table 4:** Prospective association of urinary CN1 with risk of graft failure in KTRs.

Model	Urinary CN1 Excretion		Urinary CN1 Concentration		Urinary CN1/Creatinine Ratio	
	HR [95%CI]	*p*-Value	HR [95%CI]	*p*-Value	HR [95%CI]	*p*-Value
**Model 1**	1.73 [1.44–2.08]	˂0.001	2.05 [1.69–2.49]	˂0.001	1.76 [1.48–2.09]	˂0.001
**Model 2**	1.74 [1.44–2.09]	˂0.001	2.05 [1.69–2.49]	˂0.001	1.85 [1.55–2.21]	˂0.001
**Model 3**	1.24 [1.03–1.51]	0.026	1.36 [1.11–1.67]	0.003	1.32 [1.09–1.61]	0.005
**Model 4**	1.32 [1.06–1.61]	0.011	1.43 [1.15–1.78]	0.002	1.39 [1.13–1.72]	0.002
**Model 5**	1.24 [1.01–1.52]	0.042	1.39 [1.12–1.72]	0.003	1.31 [1.06–1.60]	0.001
**Model 6**	1.23 [1.01–1.50]	0.037	1.34 [1.09–1.65]	0.005	1.31 [1.07–1.59]	0.008
**Model 7**	1.31 [1.08–1.60]	0.008	1.37 [1.12–1.69]	0.003	1.32 [1.09–1.61]	0.005

Cox regression analyses were performed to assess the associations of log2-transformed urinary CN1 excretion, urinary CN1 concentration, and urinary CN1 creatinine ratio with death-censored graft failure (number of events = 84) in KTRs (n = 703). Model 1: crude associations. Multivariable Model 2: adjusted for basic confounders (age, sex, and BMI). Multivariable Model 3 as for Model 2 with additional adjustment for eGFR and proteinuria. Subsequently, additive adjustments were performed based on variables already adjusted for in Model 3. Model 4: further adjustment for cardiovascular risk factors (systolic blood pressure, HDL cholesterol, triglycerides, antihypertensive medication usage, diabetes mellitus, and medical history of cardiovascular intervention). Model 5 involved further adjustment for transplantation-related factors (dialysis vintage, time from transplantation to baseline, donor type, need for re-transplantation up to baseline, and use of calcineurin inhibitors). Model 6: further adjusted for post-transplantation complications (delayed graft function, graft rejection, and post-transplant diabetes mellitus) and Model 7 involved further adjustment for urinary parameters (urinary sodium, urinary potassium, and urinary urea). Abbreviations: CN1: carnosinase-1, HR: hazard ratio, CI: confidence interval, eGFR: estimated glomerular filtration rate, HDL: high-density lipoprotein.

## Data Availability

The data presented in this study are available on request from the corresponding author. The data are not publicly available due to ethical reasons.

## Appendix A

### Appendix A.1. Carnosinase-1 Determination in Urine by ELISA

Highly absorbent microtiter plates (Greiner, Labortechnick, Frickenhausen, Germany) were coated overnight with goat polyclonal antihuman CN1 (1 μg/mL) (R&D, Wiesbaden, Germany). Samples were tested undiluted or in a 1:2 dilution factor. As a standard curve, serial dilutions (250–3.9 ng/mL) of mouse CN1 recombinant [His-tag] protein (Sino Biological, Beijing, China) were used. CN1 concentrations were interpolated in the linear range of the dilution curve, with a lower detection limit of 4 ng/mL. After washing, rabbit polyclonal antibody (Atlas, Abcam plc, Cambridge, UK) was added for a 1h incubation followed by goat anti-rabbit IgG antibody conjugated to horseradish peroxidase (Thermo Scientific, Eugene, Oregon, USA). BM blue peroxidase substrate (Roche Diagnostics, Mannheim, Germany) was added as a chromogen, and the reaction was stopped by the addition of 1 M H2SO4. The plates were directly read at 450 nm absorbance. Concentrations below the detection limit were set as 4 ng/mL. Urinary CN1 excretions were obtained by multiplying 24h urine volume (liters). Intra-assay coefficients of variability were 2.7% and 1.3% for concentrations of 21 ng/mL and 6 ng/mL, respectively.

### Appendix A.2. Amino Acid Profiling with Liquid Chromatography Isotope Dilution Mass Spectrometry

Samples were derivatized using the AccQTag derivatization reagent according to the manufacturer’s protocol (Waters Corporation, Milford, MA, USA) and separated using a Phenomenex Synergi™ column (4 μm Polar-RP 80 Å, 150 × 3 mm). Carnosine was detected using positive-ion electrospray ionization in multiple reaction-monitoring mode, using 13C6, 15N3-histidine as an internal standard. Data were analyzed using MultiQuant MD 3.0.2 (Sciex). Inter-assay precision was monitored using a urine pool sample. The inter-assay precisions were 7% at 22.7 μmol/L and 11% at 13.6 μmol/L. The upper limit of detection was 1200 μmol/L and values above 1200 μmol/L were reported as 1201 μmol/L. The lower limit of detection was 10 μmol/L and concentrations below were reported as 9.9 μmol/L. Urinary carnosine excretions were obtained after multiplying carnosine concentration (μmol/L) by 24 h urine volume.
